# MS-BWME: A Wireless Real-Time Monitoring System for Brine Well Mining Equipment

**DOI:** 10.3390/s141019877

**Published:** 2014-10-23

**Authors:** Xinqing Xiao, Tianyu Zhu, Lin Qi, Liliana Mihaela Moga, Xiaoshuan Zhang

**Affiliations:** 1 China Agricultural University, Beijing 100083, China; E-Mails: xxqjd@cau.edu.cn (X.X.); wingyatou@163.com (T.Z.); 2 Beijing Information S&T University, Beijing 100192, China; E-Mail: kylinx_qi@cau.edu.cn; 3 Dunarea de Jos University of Galati, 47 Domneasca St., Galati 800008, Romania; E-Mail: liliana.moga@gmail.com

**Keywords:** equipment monitoring, brine wells mining, sensor calibration, received signal strength indicator, wireless sensor network

## Abstract

This paper describes a wireless real-time monitoring system (MS-BWME) to monitor the running state of pumps equipment in brine well mining and prevent potential failures that may produce unexpected interruptions with severe consequences. MS-BWME consists of two units: the ZigBee Wireless Sensors Network (WSN) unit and the real-time remote monitoring unit. MS-BWME was implemented and tested in sampled brine wells mining in Qinghai Province and four kinds of indicators were selected to evaluate the performance of the MS-BWME, *i.e.*, sensor calibration, the system's real-time data reception, Received Signal Strength Indicator (RSSI) and sensor node lifetime. The results show that MS-BWME can accurately judge the running state of the pump equipment by acquiring and transmitting the real-time voltage and electric current data of the equipment from the spot and provide real-time decision support aid to help workers overhaul the equipment in a timely manner and resolve failures that might produce unexpected production down-time. The MS-BWME can also be extended to a wide range of equipment monitoring applications.

## 1. Introduction

Underground brine, which contains many kinds of mineral deposits such as potash, magnesium, sodium, lithium, *etc.* is an important raw material for chemical products such as salt, potash fertilizer, lithium carbonate, *etc.* [1–4]. These resources are mainly found in salt lakes around the world. The continuous chemical production plan requires ongoing brine mining, isolation and transportation [5–7]. However, the pumps are prone to easily be out of order due to the fact they must keep running round the clock in a complicated climate environment around the salt lake mining sites. The west Taijinar salt lake mine is located in Qaidam Basin, Qinghai Province, in western China. It is the largest deposit with liquid based and solid-liquid coexistence in China [8–10]. The mean annual temperature is about 3 °C, with a coldest month temperature of −12 °C and a warmest month temperature of 16 °C [11–13]. The mean annual precipitation in most of the area is below 100 mm and meteorological disasters such as strong gales, drought and hailstorms occur frequently [14–16]. Therefore it is necessary to monitor the entire running state of the pump equipment to prevent failures that may lead to unexpected production downtime with severe consequences. Literature reviews show that the voltage and electric current of the equipment can reflect its apparent running state. The equipment is working well when their values are normal and constant. On the contrary, it is abnormal when the values vary all the time or is even zero [17–19].

A number of the monitoring systems based on traditional embedded methods were developed and applied for on-site monitoring of industrial equipment. For example, a monitoring system for industrial equipment is described in [20], which is based on a low cost System-on-Chip (SoC) design using a reconfigurable hardware processing unit and a customized embedded processor. In [21], a smart sensing unit is developed for vibration measurement and machinery condition monitoring. In [22], a smart sensor system for machine fault diagnosis is presented. In [23], a power equipment monitoring system based on a wireless sensor network was designed for preventing a power system's failure. In [24], a comprehensive system for monitoring the technical state of cogeneration turbine equipment is presented. A piezoresistive micro-accelerometer with multi-beam structure was developed for vibration monitoring in intelligent manufacturing equipment in [25]. In [26], based on the technologies of condition monitoring and modern communication network, an integrated system is developed for monitoring the key running parameters of mining equipment.

However, the above adopted traditional methods are not suitable for monitoring brine well mining equipment, since the workers may have no clear information about the situation onsite. One kind of promising technology, the Wireless Sensors Network (WSN), by integrating micro-sensor technology, embedded computing, wireless communication technology with information processing, can sense and acquire information about monitored objects in their environments using sensors, record and transmit data to a base station for further processing through wireless networks [27–31]. Thus, WSNs have been applied in industry [32–34], agriculture [35–37], environment monitoring [38–40] and many other important areas.

Based on the literature review, this paper presents a novel application: a wireless real-time monitoring system (MS-BWME) that aims to monitor brine well mining equipment by acquiring and transmitting the real-time voltage and electric current data of the pump equipment from the site, helping workers deal in a timely way with the situation on the spot, overhaul the equipment, and finally resolve any failure that might produce unexpected production interruptions.

The paper is organized as follows: Section 2 presents a system analysis and architecture design, and Section 3 details the sensor nodes, network coordinator and the design and implementation of remote information management system (RIMS). System testing and evaluation are described in Section 4. Finally, the discussions and further suggestions are given in Section 5.

## 2. System Analysis and Architecture Design of MS-BWME

### 2.1. The Survey Design and Analysis

The survey adopted multiple methods to identify and extract users' requirement of the MS-BWME [41–43]. It lasted one week and five managers and fifteen workers were involved.

**Figure 1.**
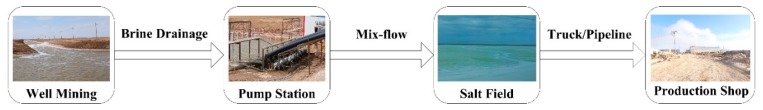
Process and information flow of the brine mining.

- Field observation for well mining and transportation to identify key monitoring point, frequency and requirement. Figure 1 describes the brine mining process in the West Taijinar salt lake mine area. The process consists of following steps:
  Step 1 Pumping the brines from the brine well. The first key monitoring points are the voltage and current of pumps, which reflect the running state of the pump equipment.
  Step 2 Transporting the mined brines to the pump station through the brine drainage.
  Step 3 Pumping the brines to the salt fields by the mixed-flow pumps in the pump station for sodium removal and enrichment of other ions such as magnesium and potassium. The voltage and current of the mix-flow pumps are another key monitoring point.
  Step 4 Generating the raw ore in the salt fields via the insolation procedure.
  Step 5 Transporting the raw ore by trucks or pipelines to the production workshop as the raw material for different chemical products such as potash fertilizer.
  Table 1 lists the monitoring parameters of key points and requirement for sensors scale and precision.
- Field survey and interviews for identifying functional requirement and system module division of the MS-BWME. An interviewee list was drawn up which included the production managers and well mining workers. They were asked to describe the work routine, how they record the information on the equipments' running state, how they handle the abnormal working status of the equipment, whether they knew about wireless monitoring or if they have used it, and what kind of requirement is expected from the system, and so forth.

### 2.2. User's Requirement of the MS-BWME

The users' requirements, extracted from the survey, are listed in Table 2 for the production managers' requirements and Table 3 for the well mining workers', respectively.

### 2.3. System Architecture Design

**Figure 2.**
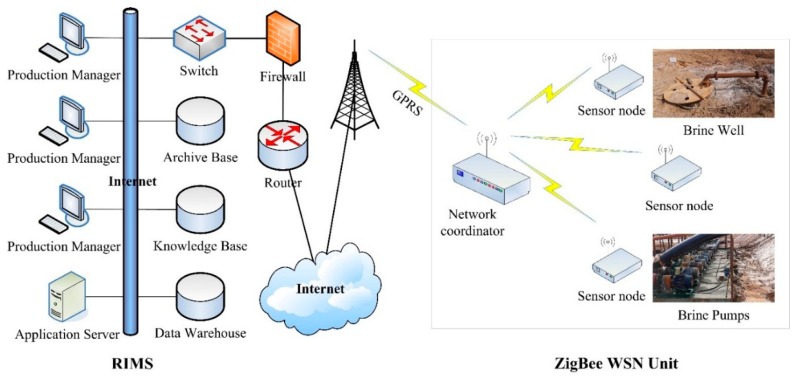
Block diagram of the MS-BWME.

The MS-BWME consists of the following units (shown in Figure 2):

- The ZigBee WSN unit is responsible for acquiring and transmitting the data, which consists of a number of sensor or router nodes and a network coordinator, and is deployed at the site of the brine well mining. The sensor nodes monitor the voltage and the electrical current of the equipment on the spot, while the network coordinator not only creates and controls the entire network, but also aggregates the sensor data from the sensor nodes. The wireless communication between the sensor node and the coordinator may be implemented directly or via the router node as a relay when they are not within an effective communication distance.
- The real-time remote monitoring unit is responsible for performing the data reception at the remote terminal. It consists of two layers: one is the sever layer, which is responsible for receiving/storing the sensor data, and serves as the pipeline to connect the users and the sensor nodes. The other is the client layer, which provides not only the real-time and reliable information for the users but also an easily used and friendly operation and configuration interface for system managers.

The function of remote wireless transmission between the ZigBee WSN unit and the remote monitoring unit is implemented via the General Packet Radio Service (GPRS). The RIMS provides functions to monitor in real-time and manage the sensor data transmitted by the GPRS remote transmission module from the coordinator. Finally, the managers at the remote monitoring center or the well mining workers can master the real-time information on the spot via the Internet and solve any failures that may produce unexpected production down-time.

## 3. System Design and Implementation of MS-BWME

This section presents more detailed information on the individual components' design and implementation of the MS-BWME.

### 3.1. Sensor Nodes Design and Implementation

#### 3.1.1. Sensor Nodes' Hardware Design and Implementation

**Figure 3.**
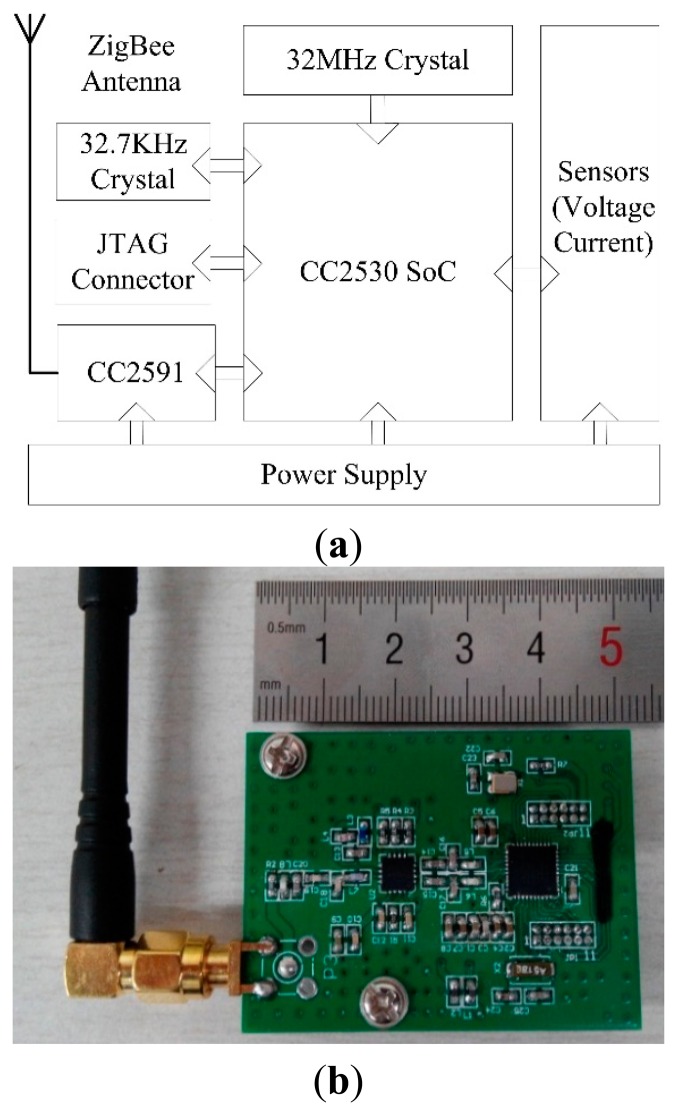
The sensor node's block diagram (**a**) and physical implementation (**b**).

The sensor node is designed as a ZigBee end-device, which integrates a microcontroller, a voltage sensor, a current sensor with a battery power supply. Figure 3a,b illustrates its block diagram and physical implementation, respectively.

- In order to improve the integration in the node and optimize the hardware design, the CC2530 wireless sensor SoC is adopted as the microcontroller, because it is a true SoC solution for IEEE 802.15.4 and ZigBee applications, integrating the excellent data measurement and processing performance of a leading RF transceiver with an industry-standard enhanced 8051 Micro Controller Unit (MCU), in-system programmable flash memory with 8-KB RAM [44].
- In order to increase the transmission distance, the CC2591 is applied as the radio frequency (RF) front end, due that it is a cost-effective and high performance RF Front End for low-power and low-voltage 2.4-GHz wireless applications [45].
- The Hall voltage sensor and the Hall current sensor is used for measuring the value of the voltage and the electrical current. Both of them are based on the principle of the Hall Effect [46–49], and a new generation industrial sensor to measure all types of voltage or current, such as alternate current (AC) and direct current (DC) with frequency of thousands Hz [50]. All of measured value output in a mode of DC voltage ranged from 0 V to 5 V Root Mean Square.
- The supply voltage of the Hall sensors is DC 24 V and dual 15 V taken from the 380 V AC power supply of the pumps; while the sensor node is supplied by a lithium battery, whose nominal voltage and capacity is 3.7 V and 1800 mAh respectively.

#### 3.1.2. Software Design of Sensor Nodes

**Figure 4.**
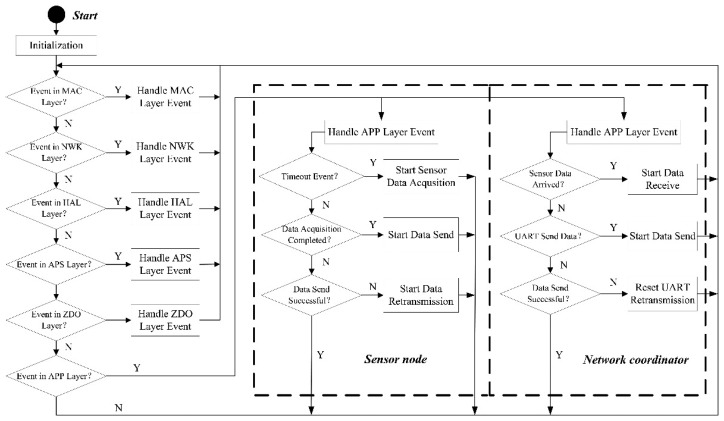
Flow chart of the sensor data acquisition at the sensor node and the data transceiver process at the network coordinator.

The sensor node embedded software is responsible for the sensor data acquisition. Its process consists of the follow steps (see Figure 4):

Step 1 Initialization of the system once the node is powered, such as the initialization of the clock, the stack and the network.
Step 2 After the initialization, the system starts the event polling and the timeout event to check whether the events response and count the time, respectively.
Step 3 The interrupt event occurs after the timeout. In the interrupt service routine, the sensor node first starts the on-chip ADC and then starts acquiring the voltage and current data.
Step 4 After the data acquisition, it calls the data request function which belongs to the Application Framework within the ZigBee protocol to send the data to the network coordinator.
Step 5 After the successful data transmission, the sensor node sleeps and doesn't wake until the next timeout. If sending the data fails, the sensor node would retransmit the sensor data.

### 3.2. Network Coordinator's Design and Implementation

#### 3.2.1. Network Coordinator Hardware Design and Implementation

**Figure 5.**
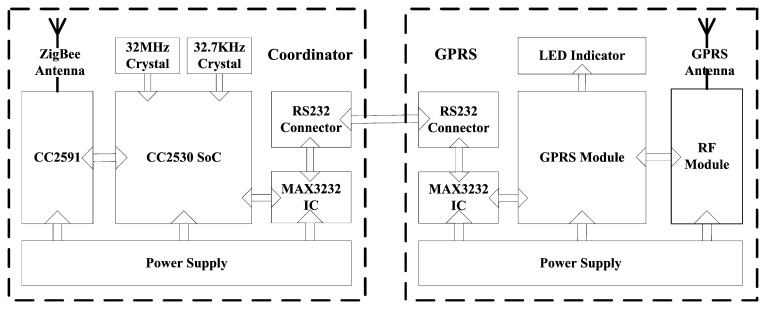
Block diagram of the network coordinator.

**Figure 6.**
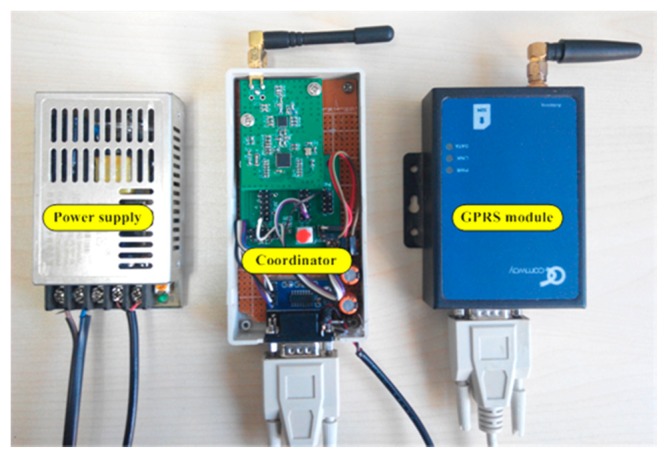
Physical implementation of the network coordinator (top view).

The coordinator adopts the CC2530 wireless sensor SoC as the microcontroller and the CC2591 as the RF front end and equips with a 5 V, 2 A power adapter to provide a continuous supply (the block diagram is shown in Figure 5). The GPRS remote transmission module is also adopted to realize the remote transmission of the aggregated sensor data. The GPRS remote transmission module is communicated with the microcontroller via the RS232 bus. The physical implementation of the network coordinator is illustrated in Figure 6.

#### 3.2.2. Network Coordinator Software Design and Implementation

The network coordinator manages the running state of the whole network. As described in Figure 4, it has similar process with sensor node. Its process consists of the follow steps:

Step 1 The network coordinator initiates the hardware and configures the RS-232 interface.
Step 2 After the initialization, the network coordinator starts a ZigBee network and waits for the network joining requests from sensor nodes.
Step 3 The network coordinator sends the synchronization instruction to all the network nodes after all nodes have joined the network.
Step 4 The network coordinator starts receiving the data and storing them into a buffer array to wait for the Universal Asynchronous Receiver/Transmitter (UART) transmission when sensor data has arrived. The coordinator resets the UART and also retransmits the sensor data when data forwarding has failed.
Step 5 The network coordinator aggregates the sensor data and performs transmission to the remote monitoring system via the GPRS module.

### 3.3. RIMS Design and Implementation

The RIMS serves as the management system for end-users, and is responsible for maintaining the database for the data acquired by the wireless sensor nodes, and provide functions to add/edit the fundamental data produced daily and search/review monitoring records.

#### 3.3.1. RIMS Architecture Design

**Figure 7.**
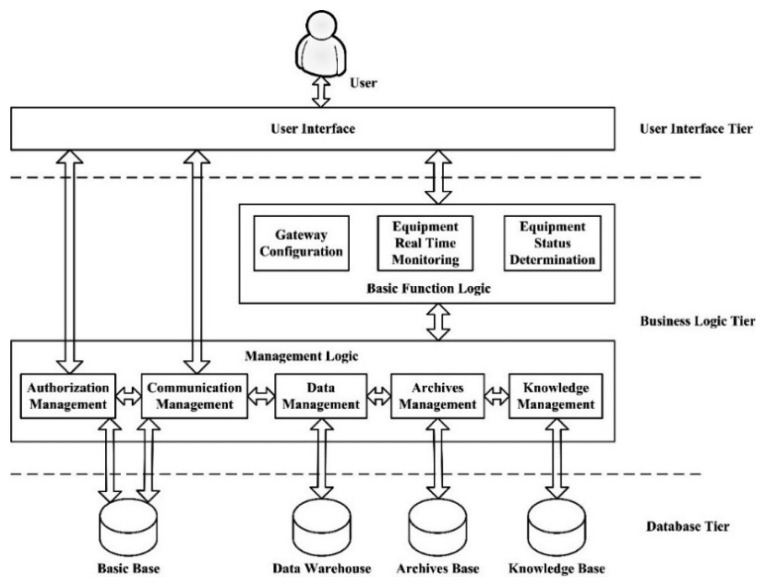
RIMS system architecture.

Based on the work flow analysis, the RIMS' functional modules, which aim to encompass the brine production cycle, adopted a 3-tier architecture, *i.e.*, User Interface tier, Functional logic tier and Database tier (shown in Figure 7).

(1) User Interface tier: provides a user interface for gateway configuration, integrity check of input data and information display, and performs data transferring between users and function logics.
(2) Business Logic tier: is responsible for a variety of processing logic calls by two components:
  - Management logic component consists of authorization management, communication management, data management, archives management and knowledge management modules. The authorization management and communication management modules exchange data with the system database in the database tier. The data management, archives management and knowledge management module exchanges data with data warehouse, archives base and knowledge base representatively.
  - Derived from the management logic, the basic function logic component consists of the gateway configuration, equipment real-time monitoring and equipment status determination. The gateway configuration module exchanges basic gateway configuration information from the communication management module, while the real-time voltage and current information exchanged between the equipment real-time monitoring module and the data management module within the management component. The component makes the selection of the equipment running statue determination model and parameters based on the knowledge management module and gets the detail information from the archives management module. When data, model and parameter are all prepared, it provides the user interface tire the detail equipment's information, the real-time voltage and current monitor information and the equipment's running status information based on the model determination.
(3) Database tier: consists of the following independent bases, which communicate with each other and are driven by representative base management module in the Business Logic tier:
  - *The basic base* is responsible for storing the authority and communication configuration information.
  - *The data warehouse* is responsible for storing the real-time voltage and current data.
  - *The knowledge base* is responsible for storing the knowledge used for the analysis and decision making.
  - *The archives database* is responsible for storing the historical data, such as well number records, pump information records, and monitoring records in brine well mining.

SQL Server 2008 database management system is applied to manage all the bases' operation.

#### 3.3.2. RIMS Implementation

**Figure 8.**
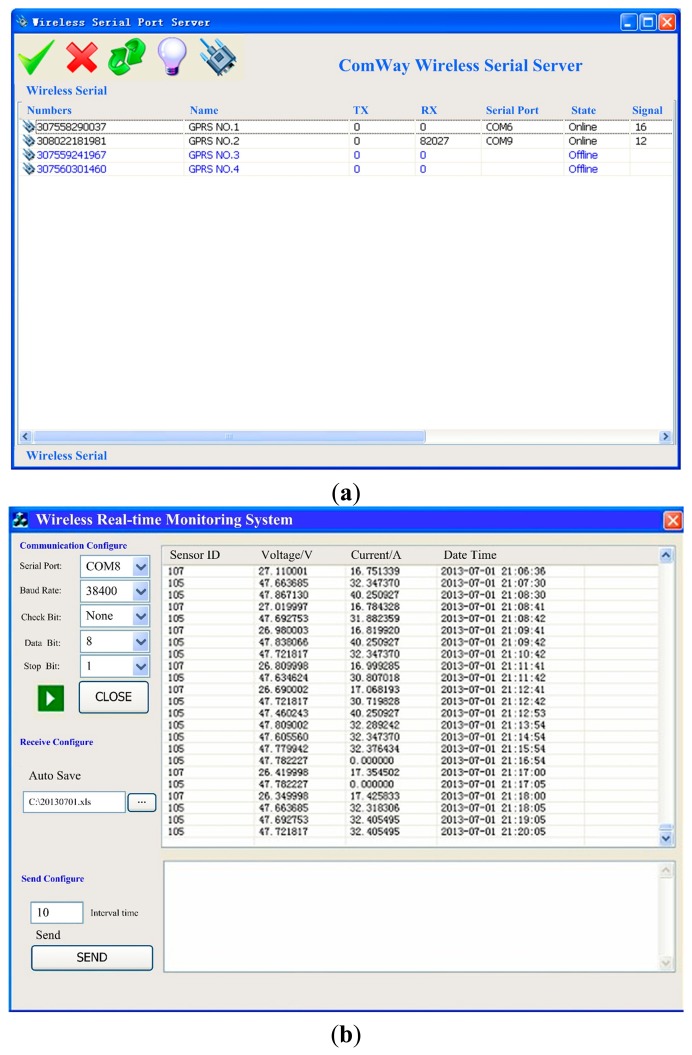
Communication configuration interface (**a**) and real-time data receiving interface in the remote monitoring center (**b**).

RIMS was developed using C# in Microsoft Visual Studio 2008 integrated with the real-time monitor chart powered by the Matlab M-language dynamic link library. The communication configuration and real-time data receiving interface of the RIMS are shown in Figure 8a which showsemonstrates the RS232 serial port configuration information and the GPRS wireless connection between the ZigBee wireless sensor network and the RIMS; Figure 8b shows the real-time data receiving interface of the RIMS. The real-time voltage and current data are displayed and stored in the database every one minute.

## 4. System Test and Evaluation of MS-BWME

### 4.1. Implementation Scenario and Experiment Design

**Figure 9.**
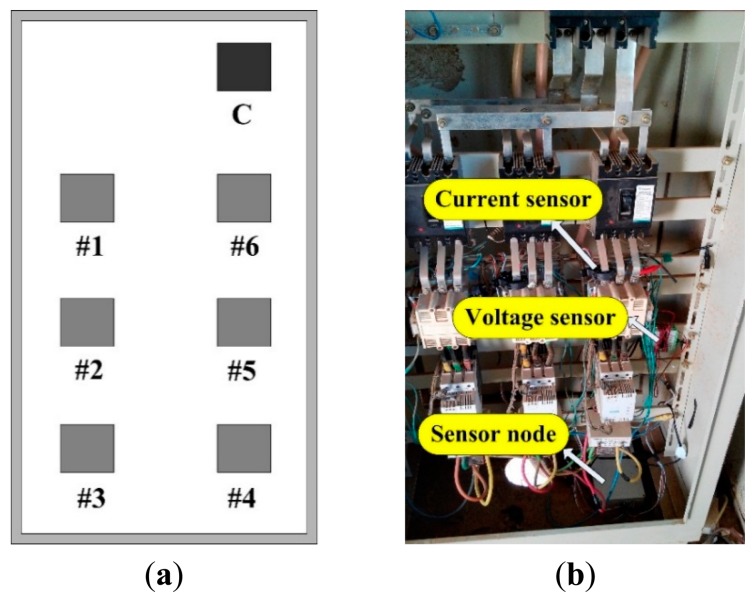
(**a**) WSN deployed in a power distribution room (15 m × 10 m) where C is the network coordinator, 1–6 are the sensor nodes; (**b**) the current sensors, voltage sensor and sensor node No. 1.

The developed WSN was deployed in the field in the brine well mining operations in Qinghai Province. It comprised six sensor nodes and a network coordinator. Figure 9a indicates the WSN deployment: the minus and maxim actual distance between the sensor nodes and network coordinator is approximately 3 m and 15 m, respectively; each node with external antenna was integrated into a plastic box and fixed at a height of approximately two meters in the distribution cabinet located in a power distribution room. Figure 9b illustrates the deployment on sensor node No. 1, the voltage sensor and current sensor. The RIMS was installed in remote control center located the company's office.

Three experiments were conducted for characterizing the performance of the MS-BWME:

- The first experiment aimed to test the performance of monitoring system data transmission according to the implementation scenario which included the sensor calibration and the system's real-time data reception.
- Then the performance of the Received Signal Strength Indicator (RSSI) between the sensor nodes and the network coordinator was focused on.
- The last experiment was supposed to check the battery status of each sensor node. The next sections describe and analyze these experimental results.

### 4.2. Evaluation on System Transmission Performance

The system transmission performance was evaluated by judging whether the sensor data were transmitted to the remote monitoring end in real-time, and whether the data reflected accurately the running state of the pump equipment. The evaluation includes sensor calibration and field tests.

**Figure 10.**
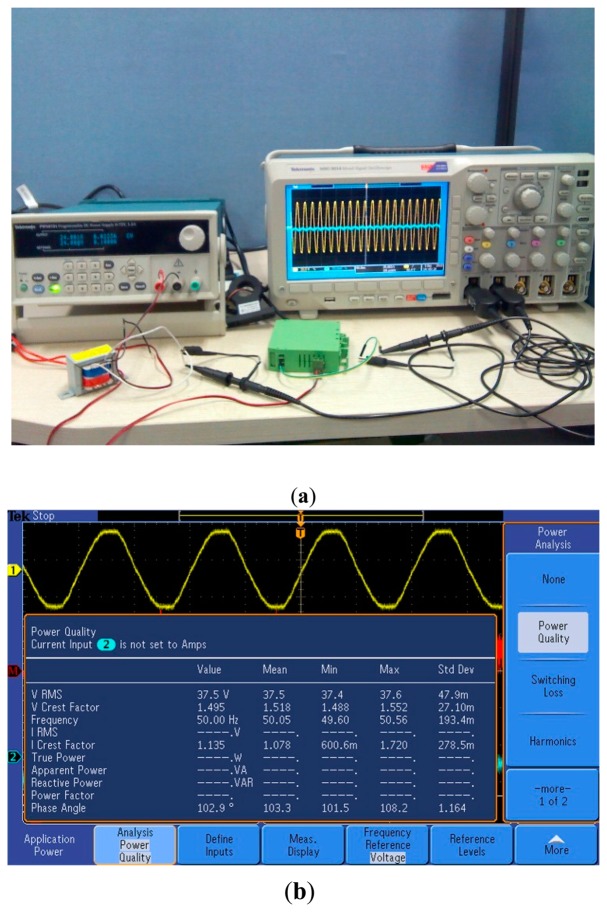
The calibration of the voltage and current sensors via the digital oscilloscope.

The voltage and current sensors calibration aims to establish the relationship between the sensors' output and input throughout the trial. It is the prerequisite for the accuracy of the measurements in actual tests. The voltage and current sensors were calibrated before deploying in the field. The absolute error of the voltage and current sensor was controlled in 0.1 V and 0.05 A, respectively. Figure 10 shows the voltage and current sensors' calibration through a digital oscilloscope. As shown in Figure 10a, the original signal was measured via the first channel, while the output signal from the sensors was measured via the second channel of the oscilloscope. The sensors were supplied with a programmable DC power supply which can provide a DC 24 V and a dual 15 V voltages. To ensure the safety of the digital oscilloscope, the 220 V AC, taken as the calibration source, was stepped-down via a transformer. The step-down signal's power quality is shown as Figure 10b. The voltage and current sensors were calibrated by comparing the output signal with the calibration source signal. The sensors' margin or zero port could be adjusted to achieve consistency with the calibration source when the sensors deviate from the calibration source.

**Figure 11.**
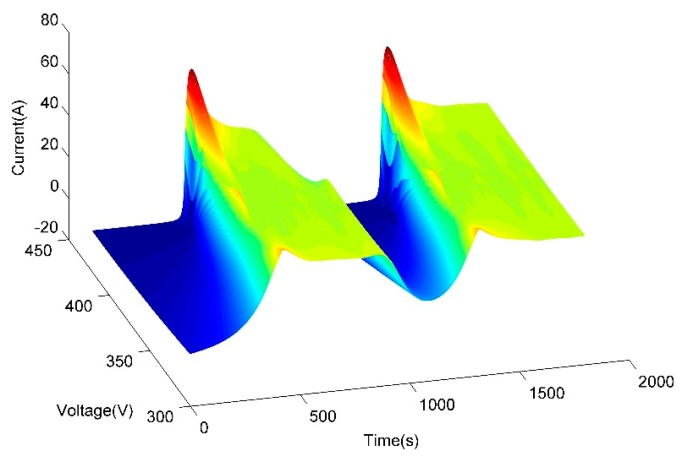
The surface chart of voltage and electrical current received during 30 min.

The sensor nodes were deployed in the field after the sensor calibration. Figure 11 shows the surface chart of the voltage and electrical current signals received from sensor No. 3 around 30 min. It indicates that the pump is out of run once during the testing period. The pump produced an instantaneous pulse current when it was started, while the pump was not started between 0 and 420 s, stopped between 900 and 1300 s and running stably during the 425–900 and 1305–1800 s.

### 4.3. Evaluation on RSSI of the Network

The link-quality indicator (LQI) and RSSI are usually adopted in the field tests to measure the wireless link quality during the operation of a network [48]. As the LQI is computed from the raw RSSI by linearly scaling it between the minimum and maximum defined RF power levels for the radio in the ZigBee protocol [51], the RSSI was measured to understand what impact it has on the physical layer of the wireless system and how it is influenced by the environmental conditions of the well mining. The RSSI was read from the received Media Access Control frame in the ZigBee protocol, which is responsible for the calculation of the RSSI value by detecting the received signal strength. The RSSI shows the received signal strength in -dBm.

**Figure 12.**
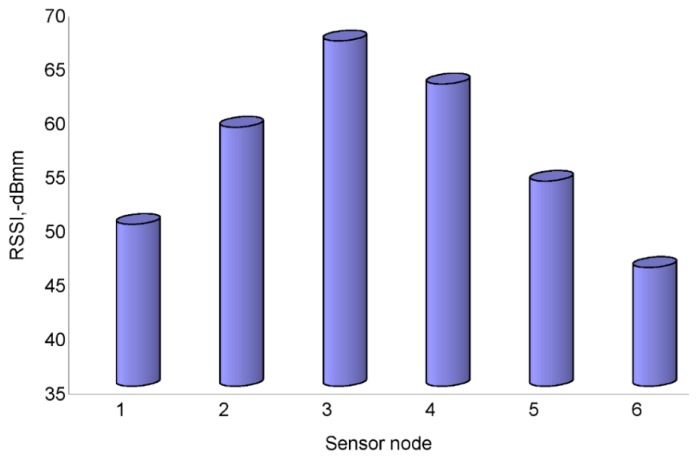
The RSSI of each link between the network coordinator and each sensor node.

The wireless links between the network coordinator and each sensor node were evaluated. For the evaluation 1000 measurements of RSSI were carried out for each link during the daytime. Figure 12 demonstrates the RSSI for each link. Both sensors No. 1 and No. 6 have a higher RSSI, while the sensors No. 3 and No. 4 are lower. The result is related to the communication distance between the network coordinator and sensor node. That the lowest RSSI is approximately −67 dBm suggests that the wireless network has a relatively reliable link on the spot.

**Figure 13.**
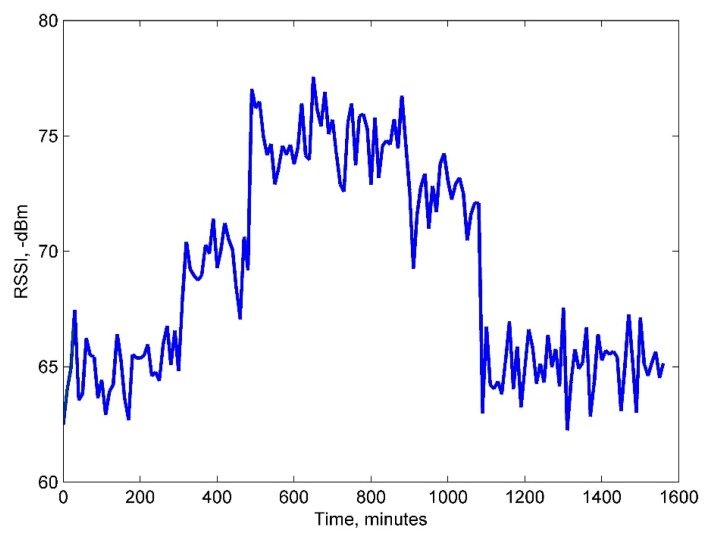
The RSSI evaluation during 1560 min (approximately 26 h).

Furthermore, the environmental impact on the RSSI stability was evaluated based on more than two weeks of monitoring data. The metric was measured between sensor node No. 3 and the network coordinator due that this sensor node has a longest distance from the coordinator and a lowest RSSI. Figure 13 shows a plot of the measurements within 24 h during the test: the RSSI is relatively stable during the daytime (0–480 min and 1200–1560 min) and shows a significant RSSI drop to around −70 dBm during the night (8 p.m. to 8 a.m. or 480–1200 min as shown in Figure 13). This suggests that environmental changes such as the dropping temperature and increasing humidity or windy weather during the nighttime have a negative influence on the RSSI performance.

**Figure 14.**
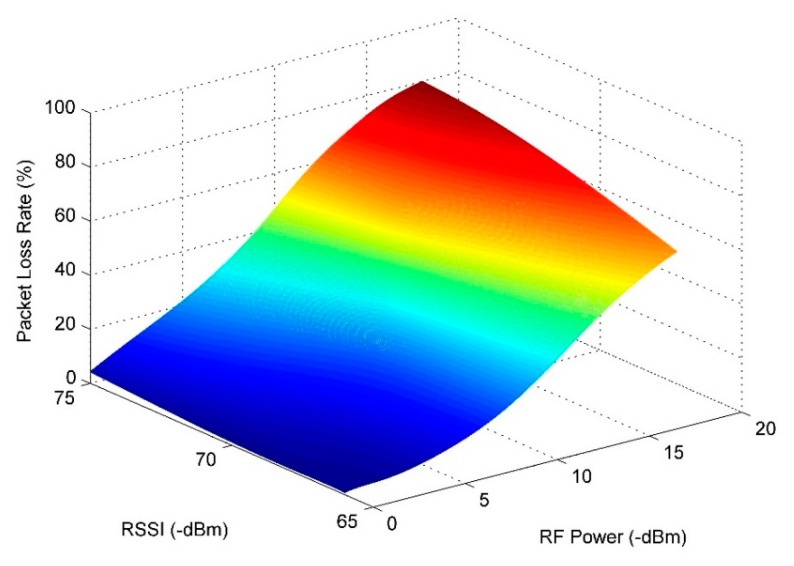
Packet Loss Rate variation with the RF Power difference and the RSSI variation.

The Packet Loss Rate from the sensor node No. 3 to the network coordinator was also evaluated. The node sent around 1000 data packets with different RF power with 10 s time intervals between submissions. Figure 14 plots the packet loss rate with the RF Power difference and the RSSI variation. It indicates the RSSI has the same change trend as the RF power: it would have a good link while the packet loss rate less than 20% or the RSSI is higher than −71.2 dBm. It is also worth noting that the packet loss rate could reach almost 0% when the RSSI is higher than −66 dBm.

Those experiments suggest that the wireless network has a relatively reliable link and transmission of the sensor data in the well mining scenario, but the stability may vary during nighttime due to environmental changes.

### 4.4. Evaluation of Sensor Nodes Lifetime

The lifetime of the sensor nodes is considered the key factor for the functioning of a WSN. Each sensor node is supplied with a 3.7 V, 1800 mAh lithium battery. The node power management circuit ensures the nodes' stable operation until the total voltage drops to 2 V (0% battery charge).

**Figure 15.**
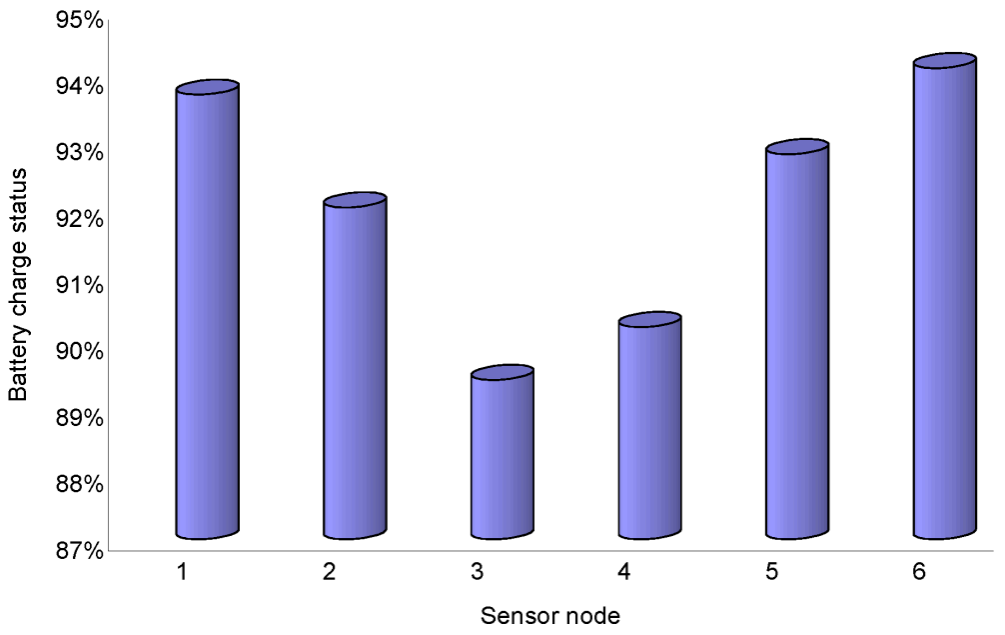
Battery charge status of the sensor nodes in the network.

Figure 15 presents the battery charge status of each sensor node after approximately one month. The measurement was performed from the beginning of June 2013 till the end of July 2013. The battery charge status varies from 89% to 94% for all nodes. The batteries of both sensor node No. 3 and No. 4 are quickly depleted because of the low RSSI link between those and the network coordinator and more power being required to establish the communication link. Based on the results of the experiment, it was predicted that the network could be in normal operation for approximately 13 months.

### 4.5. System Evaluation of the MS-BWME

System evaluation measures the improvements of brine well mining production management on technological capacity, performance and system utilization brought by the MS-BWME as well as the defects of this system prototype.

Managers and workers from the enterprise were invited to take part in the system evaluation and discuss the system performance and form a consistent view on how this system should be perfected to improve management efficiency of the brine well mining production.

Table 4 shows the performance analysis before and after the MS-BWME implementation; Table 5 shows the suggestions for the MS-BWME improvement and perfection.

## 5. Conclusions

This paper presents a novel application of the MS-BWME, which is implemented and evaluated in an actual production field for monitoring the brine well mining equipment in Qinghai, China.

The system test and evaluation show that MS-BWME can monitor the running state of the pump equipment of the brine well mining operation by acquiring and transmitting the real-time voltage and electric current data of the equipment from the spot, helping workers overhaul the equipment in a timely fashion and resolve any failures that might cause unexpected production down-time.

MS-BWME can transmit the sensed data to the remote monitoring center real-time via the WSN and GPRS remote transmission module from the spot and reflect accurately the running state of the pump equipment. At the same time, the wireless network has a relatively reliable link in the well mining scenario although while it may display reliable transmission of the sensor data during the daytime, and the RSSI of sensor node No. 3 was approximately −67 dBm, it may fail due to the low RSSI at night, when the RSSI of sensor node No. 3 was around −70 dBm. Finally, the network was estimated to be able to operate successfully for approximately 13 months in total by checking the battery charge status of each sensor node.

The link quality between the sensor node and the network coordinator is influenced by the environmental changes, such as temperature or humidity changes, and windy or rainy weather. Therefore, the on-site immunity and stability of the MS-BWME should be improved.

The MS-BWME can be extended to a wide range of equipment monitoring applications. Meanwhile, it is necessary to integrate low energy sensors into the sensor nodes and the network coordinator to meet the demands of scable application.

## Figures and Tables

**Table 1.** The well mining equipment monitoring parameters.

**Parameter**	**Equipment Ranges**	**Sensor Module**	**Sensor Range**	**Sensor Accuracy**
Voltage (AC)	0–380 V	CHS-500	0–500 V	± 0.5%FS
Current (AC)	0–48 A	A-CS050EK	0–50A	± 0.5%FS

**Table 2.** Production managers' requirements for MS-BWME.

**Requirement ID**	**Requirement Type**	**MS-BWME Should:**
Req1	Functional	Enable the managers to acquire the real-time information on the well mining pumps' voltage in the remote monitoring center.
Req2	Functional	Enable the managers to obtain timely information on the well mining pumps' electrical current in the remote monitoring center.
Req3	Functional	Enable the managers to obtain timely information on the running state of the well mining pumps in the remote monitoring center.
Req4	Non-Functional	Be easily operated by the end users.

**Table 3.** Well mining workers' requirements for MS-BWME.

**Requirement ID**	**Requirement Type**	**MS-BWME Should:**
Req5	Functional	Be a running state indicator showing the well mining pumps' voltage and electrical current information to the workers on the spot.
Req6	Functional	Be a flexible running state indicator that could be easily adopted to other kinds of well mining equipments via a simple system configuration.
Req7	Non-Functional	Be convenient to equipment on the spot and easy to use.

**Table 4.** Performance analysis before and after the MS-BWME implementation.

**ID**	**Content**	**Before Implementation**	**After Implementation**
1	Pumps' remote voltage monitoring	Null	Real-time
2	Pumps' remote current monitoring	Null	Real-time
3	Pumps' running state remote monitoring for production managers	Null	Real time
4	Pumps' running state on-site indicating for well mining workers	Null	Real time

**Table 5.** Suggestions for the improvement and perfection of MS-BWME.

**ID**	**Suggestion**	**Suggestion Type**
1	Increase the WSN immunity and stability on-site	Functional
2	Reduce the economic costs of sensor nodes	Non-functional
3	Integrate the network coordinator	Non-functional
4	Integrate the low energy sensors into the sensor nodes	Non-functional
